# Insomnia as an Unmet Need in Patients With Chronic Hematological Cancer: Protocol for a Randomized Controlled Trial Evaluating a Consumer-Based Meditation App for Treatment of Sleep Disturbance

**DOI:** 10.2196/39007

**Published:** 2022-07-01

**Authors:** Jennifer Huberty, Nishat Bhuiyan, Ryan Eckert, Linda Larkey, Megan Petrov, Michael Todd, Ruben Mesa

**Affiliations:** 1 Science Calm San Francisco, CA United States; 2 College of Health solutions Arizona State University Phoenix, AZ United States; 3 Mays Cancer Center University of Texas Health San Antonio San Antonio, TX United States; 4 Edson College of Nursing and Health Innovation Arizona State University Phoenix, AZ United States

**Keywords:** hematological cancers, mobile health, mHealth, meditation, sleep disturbance, mobile phone

## Abstract

**Background:**

To address the need for long-term, accessible, nonpharmacologic interventions targeting sleep in patients with chronic hematological cancer, we propose the first randomized controlled trial to determine the effects of a consumer-based mobile meditation app, Calm, on sleep disturbance in this population.

**Objective:**

This study aims to test the efficacy of daily meditation delivered via Calm compared with a health education podcast control group in improving the primary outcome of self-reported sleep disturbance, as well as secondary sleep outcomes, including sleep impairment and sleep efficiency; test the efficacy of daily meditation delivered via Calm compared with a health education podcast control group on inflammatory markers, fatigue, and emotional distress; and explore free-living use during a 12-week follow-up period and the sustained effects of Calm in patients with chronic hematological cancer.

**Methods:**

In a double-blinded randomized controlled trial, we will recruit 276 patients with chronic hematological cancer to an 8-week *app-based wellness* intervention—the active, daily, app-based meditation intervention or the health education podcast app control group, followed by a 12-week follow-up period. Participants will be asked to use their assigned app for at least 10 minutes per day during the 8-week intervention period; complete web-based surveys assessing self-reported sleep disturbance, fatigue, and emotional distress at baseline, 8 weeks, and 20 weeks; complete sleep diaries and wear an actigraphy device during the 8-week intervention period and at 20 weeks; and complete blood draws to assess inflammatory markers (tumor necrosis factor-α, interleukin-6, interleukin-8, and C-reactive protein) at baseline, 8 weeks, and 20 weeks.

**Results:**

This project was funded by the National Institutes of Health National Cancer Institute (R01CA262041). The projects began in April 2022, and study recruitment is scheduled to begin in October 2022, with a total project duration of 5 years. We anticipate that we will be able to achieve our enrollment goal of 276 patients with chronic hematological cancers within the allotted project time frame.

**Conclusions:**

This research will contribute to broader public health efforts by providing researchers and clinicians with an evidence-based commercial product to improve sleep in the long term in an underserved and understudied cancer population with a high incidence of sleep disturbance.

**Trial Registration:**

ClinicalTrials.gov NCT05294991; https://clinicaltrials.gov/ct2/show/NCT05294991

**International Registered Report Identifier (IRRID):**

PRR1-10.2196/39007

## Introduction

### Symptom Burden and Sleep Disturbance Among Patients With Chronic Hematological Cancer

Hematological cancers, a group of cancer subtypes that include blood- and lymph-related disorders, account for 11% of all cancer diagnoses in the United States [1], with an estimated 1.9 million patients with hematological cancer currently living in the United States [1]. New noncurative therapies have led to improved survival for many hematological cancers; however, these maintenance medicines leave patients in a state of chronic disease. Approximately 60% of chronic hematological cancers, which include chronic leukemias (chronic lymphocytic and chronic myelogenous leukemia), low-grade lymphomas (follicular lymphomas), myeloma, myelodysplastic syndrome, and myeloproliferative neoplasms (MPNs), are incurable and typically follow unpredictable remitting-relapsing pathways associated with varying needs for treatment, which may be distressing for patients [2]; thus, these patients have distinct needs from those of patients with solid tumors (eg, breast cancer) [3]. Patients with chronic hematological cancers experience ongoing disease-induced symptoms that are frequently not resolved by medical therapy and chronic maintenance therapy-associated toxicities. This chronic symptom burden (eg, sleep disturbance, inflammation, fatigue, and emotional distress) persists into survivorship and re-emerges over time, despite the best available pharmacological therapy [1].

In particular, sleep disturbance (eg, difficulty falling asleep, difficulty staying asleep, nonrestorative sleep, or insomnia disorder) [4] is highly prevalent among patients with chronic hematological cancer [5]. Across studies, 50% to 80% of patients with hematological cancer have reported sleep disturbance [6-8]. Findings for the treatment of sleep disturbance in other highly studied cancer populations, such as breast cancer, may not be generalizable to patients with chronic hematological cancer, given their unique needs and disease course [9,10]. There is a pressing need for evidence-based, accessible treatments to improve *sleep* specifically in patients with chronic hematological cancers.

In addition, in patients with chronic hematological cancer, sleep disturbance is a predictor of increased inflammatory cytokine levels [10,11]; increased fatigue [10]; and poorer emotional functioning or emotional distress [10], including depressive symptoms [10]. In both noncancer and solid tumor cancer populations, sleep disturbance increases inflammation through persistent activation of the hypothalamic-pituitary-adrenal axis, which can, in turn, induce glucocorticoid resistance in immune cells, ultimately leading to a state of chronically elevated proinflammatory cytokines [12]. Sleep disturbance has also been shown to induce fatigue [13,14] and emotional distress [15-17]. The joint occurrence of sleep disturbance, fatigue, and emotional distress is referred to as symptom clustering [18] or grouping of interrelated symptoms. Such clusters have been shown to have a negative impact on patient survival [14,19]. These factors are also known to interact in complex ways, potentially leading to negative feedback loops that further affect symptom clustering (eg, as inflammation increases, further decrements in sleep and fatigue may be observed) [20].

### Current Approaches to Addressing Sleep Disturbance

Medications are the most commonly prescribed treatment for patients with cancer experiencing sleep disturbances; however, to date, there is insufficient evidence to conclude that they are truly effective, particularly when considering long-term treatment of sleep problems [4,21]. In addition, medication may exacerbate symptoms associated with chronic hematological cancer, such as increased severity of insomnia, fatigue, and pain [22]. Potential deleterious side effects, along with the poor long-term success of pharmacological treatment for managing sleep disturbance, may outweigh any potential effectiveness for improving sleep and related quality of life in patients with chronic hematological cancer, indicating a need for alternative, safe, and effective sleep management strategies.

Nonpharmacologic behavioral interventions are promising strategies for treating sleep disturbance because of their (1) limited side effects, (2) targeting of specific psychological and behavioral factors that interfere with sleep, and (3) potential for long-lasting effects [23]. Currently, cognitive behavioral therapy for insomnia (CBT-I) is the most widely available, evidence-based, nonpharmacological treatment strategy for managing sleep disturbances in patients with and survivors of cancer [24]. However, CBT-I is intensive and time consuming for patients, requiring frequent in-person meetings with therapists and physicians at sites or clinics [21]. Remote delivery of CBT-I may be an alternative approach; however, studies assessing remotely delivered CBT-I have not been conducted for patients with cancer [21]. Even app-based CBT-I requires considerable effort and time to be effective [21]. Specifically, cognitive behavioral therapy apps require both clinician involvement and support, along with high adherence on the patient’s side, to be effective [25]. Patients with chronic hematological cancer need less burdensome intervention strategies for long-term sleep management. Currently, patients with chronic hematological cancer have limited options for easily accessible, evidence-based treatments for sleep disturbance, exacerbating the diminished quality of life and resulting in poorer cancer outcomes.

### Evidence and Previous Literature Supporting Meditation

Meditation is a sleep disturbance treatment strategy recommended by the American Cancer Society and the National Center for Complementary and Integrative Health. Mindfulness meditation is the practice of moment-to-moment awareness, in which a person purposefully focuses on the present without judgment [26]. Several reviews and meta-analyses have demonstrated the positive effects of mindfulness-based interventions for general sleep complaints and sleep parameters associated with sleep quality (eg, total sleep time, sleep efficiency, and sleep onset latency) [27-29], yielding moderate effect sizes comparable with those of standard of care treatments for insomnia [28]. Individual studies have also demonstrated effects on specific sleep disorders, including insomnia [30], narcolepsy [31], restless leg syndrome [32], and sleep apnea [33]. Ong et al [34] suggested that meditation promotes balanced appraisals, cognitive flexibility, equanimity, and recommitment to values, which can, in turn, allow sleep-related arousal to subside and normal sleep patterns to re-emerge. Shallcross et al [27] further posited that sleep disturbance involves five processes: (1) rumination, (2) primary arousal, (3) secondary arousal, (4) sleep monitoring and selective attention and effort, and (5) distorted perceptions. The core processes involved in mindfulness (ie, experiential awareness, attentional control, and acceptance) have the potential to target each of the 5 processes contributing to sleep disturbance [27].

Meditation also has a dose-response relationship with reductions in inflammation for both those with solid tumors and those without cancer [35-37]. One review found that mindfulness-based interventions had no effect on cytokines in healthy populations but improved cytokine levels in patients with cancer [38]. Mindfulness-based interventions aid the recovery of the immune system, and the resulting immune changes show a shift from a carcinogenic cytokine profile to a more normalized profile. Meditation and other mindfulness-based practices have improved inflammatory biomarker levels common in patients with chronic hematological cancer (eg, tumor necrosis factor-α [TNF-α], interleukin [IL]–6 [IL-6], IL-8, and C-reactive protein [CRP]), as well as in populations such as patients with insomnia disorder, survivors of cancer, and older adults [39-42]. These specific cytokines and proteins are the most common inflammatory cytokines closely associated with chronic hematological cancer disease progression, symptoms, and sleep disturbances across all hematologic malignancy subtypes [11,43,44]. The calming effects of meditation on the autonomic nervous system and the hypothalamic-pituitary-adrenal axis may reduce proinflammatory gene expression and signaling via the downregulation of the proinflammatory transcription factor necrosis factor κβ pathway [35,45].

Meditation has previously demonstrated improvements in both sleep disturbance and fatigue among a subset of patients with chronic hematological cancer (MPN), with patients reporting that their reductions in fatigue were because of better sleep quality [46,47]. Several other studies have established the effects of mindfulness interventions in reducing fatigue in patients with cancer [48-50]. For example, Ikeuchi et al [51] found that mindfulness affected fatigue both directly and indirectly through anxiety, depression, pain, loneliness, and sleep disturbances. Randomized controlled trials (RCTs) have also shown that meditation reduces sleep disturbance by reducing emotional distress (eg, anxiety and depression) in patients with and survivors of cancer [52] and reducing sleep-interfering cognitive processes (eg, ruminating thoughts) [53,54]. Mindfulness meditation may reduce stress, worry, intrusive thoughts, and presleep arousal, which may, in turn, reduce sleep disturbance [55]. In particular, patients with chronic hematological cancer are more likely to experience such issues as their disease is never cured.

Although the Society for Integrative Oncology Clinical Practice Guidelines [56] suggests that meditation is a safe technique for decreasing the burden of a variety of cancer-related symptoms and comorbidities, few studies have investigated the effects of mindfulness meditation on sleep disturbance, specifically in patients with chronic hematological cancer [57]. In 2 recent RCTs with patients with leukemia, meditation interventions were delivered in person in hospital settings by trained clinicians [58,59], thereby limiting accessibility and use, especially in patients who were fatigued, had inflexible schedules, or had transportation difficulties [60]. Traditional in-person meditation programs for patients with cancer are lengthy and potentially burdensome (eg, 30- to 60-minute sessions on a single day of the week [58]), especially for patients with chronic hematological cancer and high chronic symptom burden [61]. Of note, meditation studies with patients with leukemia had short durations (only 5 weeks in length), were conducted at a specific time during treatment and in a clinical inpatient setting (eg, during chemotherapy treatment sessions [58] or in the hospital [59]), and did not measure sustained practice or effects outside of the clinic setting. Prior studies of meditation in limited cancer settings have supported extending and testing flexible and effective modes of delivering meditation to understudied groups with cancer.

### Mobile App Use Among Patients With Chronic Hematological Cancer

Research further demonstrates that patients with chronic hematological cancer are interested in smartphone apps as a means of self-managing their health [38-40]. As of April 2020, there were approximately 55 mobile apps marketed to patients with or survivors of cancer available in the Google Play and Apple App stores, offering mostly educational content and information for caregivers. Of these, 4 included some form of meditation. No commercially available meditation apps for cancer populations have been rigorously evaluated for their feasibility, safety, or effectiveness [41,42]. A review identified only 2 meditation studies for patients with cancer delivered via mobile apps; both focused on breast cancer, and only 1 tested a consumer-based app [62]. In one of these studies, an adapted mindfulness-based stress reduction (MBSR) program was delivered via an iPad, resulting in significant improvements in psychological status and quality of life among MBSR participants compared with a usual care control group [63]. However, this study was conducted over 6weeks without any follow-up [63]. The other study assessed a consumer-based app, Headspace, and found an increased quality of life and mindfulness in Headspace participants compared with the control group but did not obtain app use data in most participants [64]. Meinlschmidt et al [65] suggested that there is more value in identifying and testing popular and currently available tools that have a committed user base and are likely to remain in use in the near future. To date, few interventions have tested the effects of consumer-based, commercially available meditation apps for sleep, and none have been tested to reduce sleep disturbances in chronic hematological cancer. A summary of key takeaways from current literature in this area is presented in Textbox 1.

**Key takeaways from current literature.**

**Key points regarding sleep disturbance in chronic hematological cancer**

- Sleep disturbance is a long-term and serious problem for patients with hematological cancer and warrants distinct treatment.
- Patients with chronic hematological cancer lack effective, long-term treatment options for sleep disturbance.
- Mindfulness meditation has been rigorously tested as a nonpharmacological complementary therapy that leads to the amelioration of sleep disturbances and specific sleep disorders.
- Mindfulness meditation consistently improves factors associated with sleep disturbance in populations with cancer: inflammation, fatigue, and emotional distress.
- Current meditation interventions do not meet the diverse and unique needs of patients with chronic hematological cancer; more flexible options are needed.
- Consumer-based apps have great potential to deliver meditation interventions; however, more rigorous studies are needed.

### This Trial

Therefore, leveraging a unique academic-industry partnership, we propose a 20-week RCT (8-week intervention+12-week follow-up) to rigorously test the consumer-based app Calm to effectively deliver a meditation intervention to patients with chronic hematological cancer (N=276; 138 per group) as compared with a health education podcast control group. Calm was chosen for this study as our research team had rigorously tested the app among patients with chronic hematological cancer previously, and we have demonstrated its preliminary efficacy and preference among patients with chronic hematological cancer over another popular meditation app, Headspace [47]. This study aims to (1) test the efficacy of daily meditation (≥10 minutes per day) delivered via Calm compared with a health education podcast control group in improving the primary outcome of self-reported sleep disturbance, as well as secondary sleep outcomes, including sleep impairment and sleep efficiency; (2) test the efficacy of daily meditation (≥10 minutes per day) delivered via Calm compared with a health education podcast control group on inflammatory markers (TNF-α, IL-6, IL-8, and CRP), fatigue, and emotional distress (anxiety and depressive symptoms); and (3) explore free-living use during a 12-week follow-up period and sustained effects at 20 weeks from the baseline of Calm in patients with chronic hematological cancer.

## Methods

### Ethics Approval

All study procedures and materials were approved by the Biomedical Research Alliance of New York Institutional Review Board (study 21-136-955) and conducted in accordance with the Declaration of Helsinki. This study has been registered at ClinicalTrials.gov (NCT05294991). Written informed consent will be obtained from all participants.

### Study Design

This study is a double-blind RCT in which eligible and consenting study participants will be randomly assigned to one of two groups: (1) the daily Calm meditation intervention group or (2) health education podcast control group.

### Recruitment

Patients with chronic hematological cancers will be nationally recruited using previously established organizational partners (eg, Leukemia and Lymphoma Society, MPN Research Foundation, Patient Empowerment Network, Banner Health, and Mays Cancer Center at UT Health San Antonio MD Anderson). Recruitment materials will be developed specifically for each organizational partner to advertise the study to potential participants via social media (eg, Facebook, Instagram, and Twitter), email listservs, website postings, and postings in clinics or waiting rooms. The study will be advertised as an *app-based wellness* study to blind study participants to the intended Calm intervention group.

### Enrollment and Randomization

Interested patients with chronic hematological cancer will be directed to complete a web-based eligibility screening questionnaire via REDCap (Research Electronic Data Capture; Vanderbilt University; see Textbox 2 for a detailed list of the eligibility criteria). Ineligible participants will be sent an email notifying them of their ineligibility status and providing resources for patients with cancer, which will include an educational sleep handout and a discount code for Calm. Eligible participants will be sent an email notifying them of their eligibility; asked to review a video-based overview of the study (including the informed consent); and sent a link to electronically sign the informed consent via REDCap, a secure web-based data collection and management platform. Participants will be offered the option of scheduling a call with a member of the research team to review any questions related to the study before providing their consent. Once consent is signed, participants will be (1) asked to complete the baseline questionnaire via REDCap, (2) blindly and randomly assigned to either the Calm intervention or health education podcast control arm, and (3) emailed the study materials (eg, app download instructions, blood draw instructions, and welcome email with important dates and study milestones). As part of the study instructions, all participants will be asked not to participate in any similar interventions, including other meditation apps, medications, or nonpharmacological sleep interventions during the study period. Participants will be blocked or stratified by biological sex and then randomly allocated (with a 1:1 allocation ratio) within the male-female strata to treatments using a pseudorandom number algorithm provided by the study statistician. Research personnel who assign participants to a group will not be the same as those who enroll participants or download and clean data. The group allocation status will be restricted so that statisticians and researchers will remain blind to treatment assignment.

After randomization, all participants will be offered an app download coaching and tutorial call with a study team member to review their assigned app, including a review of (1) the general app layout and basic functionalities (eg, look, feel, and types of content found on the app), (2) where to find content and how the content is organized, (3) how to view participation-tracking statistics, (4) how to set reminders to meditate or view podcasts, and (5) how to share participation statistics on social media. Following the call, the participants will receive an email containing a phone number for continued support using the app.

In addition, the participants will receive blood draw instructions. Participants will have a blood draw request placed to obtain a blood draw from their local Quest Diagnostics Patient Service Center and instructed on the time of day to receive their blood draw and the forms of identification they will need to obtain the blood draw. Participants will have access to a study team member’s phone number and email for further support while receiving a blood draw throughout the study.

Finally, participants will receive a mailed package containing (1) an Actiwatch Spectrum device; (2) instructions detailing the wear, care, and use of the device; and (3) instructions for shipping the device back to the research team at the study conclusion. The participants will not proceed with the intervention until the previous steps are fully completed.

**Study eligibility criteria.**

**Inclusion criteria**

- Diagnosis of chronic hematological cancer on stable maintenance management (chronic hematological cancer subtypes include: chronic lymphocytic leukemia, follicular lymphoma, smoldering myeloma, myeloma, myeloproliferative neoplasm subtypes [polycythemia vera, essential thrombocythemia, and myelofibrosis], systemic mastocytosis, chronic myelomonocytic leukemia, and myelodysplastic syndrome) by a treating physician:
- On stable medical therapy or observation (ie, no changes in disease-targeted medications [or their dose] for the past 2 months)
- Not currently participating in a therapeutic pharmacologic clinical trial
- Has not received and is not planning to receive an allogeneic stem cell transplantation
- Self-identifying as sleep disturbed (Pittsburgh Sleep Quality Index score of >5, indicating moderate sleep disturbance)
- Owning and able to operate a mobile smartphone (iPhone with iOS 9.0 or later or an Android 4.1 or later)
- Willing to download and able to operate a mobile app
- Able to understand written and spoken English
- Aged ≥18 years
- Willing to be randomized
- Willing to drive to Quest Diagnostics Patient Service Center 3 times during the study in the morning
- No change in sleep medication use (if any) over the past 6 weeks

**Exclusion criteria**

- Meditation or meditative movement practice (ie, yoga, tai chi, and qi gong) of ≥60 minutes per month in the past 6 months
- Use of any consumer-based meditation app
- Residing outside of the United States
- Any planned change in pharmacological therapy (ie, new drug) during the study time frame (ie, 20 weeks)
- Self-reporting sleep-disordered breathing or sleep movement disorder
- Taking sleep medications or supplements ≥3 times per week
- Self-reporting any other diagnosed and uncontrolled medical or psychiatric condition

### Intervention Group (Calm)

Calm does not provide its users with a specific prescription for how often to use the app (frequency) or how long to use the app (dose); users self-select the frequency, dose, and timing of engagement, as well as which content and features to use. The Calm intervention participants will be asked to complete a minimum of 10 minutes per day of meditation via Calm, at any time of day for 8 weeks. During weeks 1 to 4, participants will be asked to complete the introductory 30-day program *How to Meditate* comprising 30 days of 10-minute daily meditation along with education about what mindfulness is, how to practice and achieve it with meditation, the seated or reclining positions to use for meditation, and how to monitor and guide their breathing. During weeks 5 to 8, participants will be asked to complete one daily meditation that may include the *Daily Calm*, a different 10-minute mindfulness meditation each day, or a meditation from various series of meditations (eg, loving-kindness and 7 days of managing stress). Participants will have complete autonomy over choosing the meditation, which includes options for at least 10 minutes (meditation periods are tracked). After completing their daily meditation, participants will also be able to use the app content more broadly (ie, nonmeditative content, including Sleep Stories and Daily Move) based on their preference.

Calm meditation focuses on mindfulness components [66], breathing techniques, and body scans, all of which are consistent with core mindfulness practices, such as MBSR (nonjudgmental moment-to-moment awareness) [67] and one of Vipassana’s principles—objective observation of physical sensations in the body [68]. As an ideal dose for mindfulness meditation interventions has not been established because of considerable variability in intervention prescription [69], the 10-minute daily dose was chosen, as Calm currently offers 10-minute meditations (daily and series meditations), which corresponds to how long users typically meditate using the app. Practical guides recommend that beginners start with short daily meditations lasting between 10 and 30 minutes per session [26,70]; thus, the intervention prescription will be a minimum of 10 minutes per day of meditation to be effective but not overly burdensome.

Participants will be allowed to choose the time of day when they meditate. Giving participants autonomy over choosing the time of day for meditation mirrors the way in which Calm’s paying subscribers use the app, thus yielding benefits (eg, ecological validity and participant satisfaction) that likely outweigh those of imposing strict controls over the timing of use, which could lead to lower adherence (eg, missed or truncated practice). Participation (meditations completed, time of day, length of time, and nonmeditative content used) will be tracked using the Calm app throughout the study and addressed in sensitivity analyses.

After completion of the 8-week study intervention period, the Calm intervention participants will no longer have to complete daily meditations but will continue to have access to the app for 12 additional weeks during the follow-up period. Participation in the Calm app will continue to be tracked during this time.

### Control Group (Health Education Podcast)

Similar to the Calm intervention group, podcast group participants will listen to a minimum of 10 minutes per day of a health education podcast delivered via a mobile app for 8 weeks (Tables 1 and 2). A library of health education podcasts will be available on the app, with carefully chosen content that excludes any recommendations for sleep, meditation, or mindfulness-like principles or practice. Participants will be able to select the podcast based on title, time, or both in an effort to mirror the delivery of meditations in the Calm intervention group. Podcast group participants will also be encouraged to use other components of the podcast. Participation (podcasts completed, time of day, length of time, and components used) will be tracked on the app throughout the study period. After completion of the 8-week sham intervention period, the podcast group participants will no longer be asked to listen to daily podcasts but will continue to have access to the app for 12 additional weeks during the follow-up period. Participation in the podcast app will still be tracked during this period. Upon completion of the entire 20-week study period, podcast group participants will be given a discount code for 6 months of free access to the Calm app.

**Table 1:** Calm and podcast group prescriptions.

Week	Calm intervention group	Podcast group^a^
Week 1: total 7 days	Daily introductory 10-minute meditation	Daily 10-minute health education podcasts
Weeks 2-4: total 21 days	Daily 10-minute exploration of mindfulness meditation	Daily 10-minute health education podcasts
Weeks 5-20: Full access	Full library of individual and series of 10-minute meditations	Full library of podcasts available

^a^Podcasts will be uploaded weekly, except during weeks 5-20 when the remaining full library will be uploaded.

**Table 2:** Examples of Calm and podcast group prescriptions.

Group and examples			Minutes
**Ca** **lm intervention group**			
	Returning to the Here and Now	10	
	Acceptance	11	
	Paying Attention	10	
**Podcast group**			
	Alcohol and Cancer	10	
	Community Gardening	10	
	10 Ways to Have a Better Conversation	12	

### Retention and Adherence Strategies

We will use self-determination theory (SDT) and SDT-based strategies to guide our protocol and promote adherence [71]. The SDT postulates that the initiation and maintenance of behavior is influenced by three basic needs:(1) competence, or the need to produce desired outcomes and to experience mastery; (2) autonomy, or the need to feel ownership of one’s behavior; and (3) relatedness, or the need to feel connected to others [72]. Participants will be encouraged to use the features built into the Calm intervention or podcast, which are designed to help maintain engagement and adherence in their assigned app. All these features are consistent with practices that help individuals participate in health behaviors [71], and these features are identical in both apps. To promote autonomy, a feature of the Calm and podcast apps includes time spent meditating or listening to podcasts—participants can go to their Calm profile and they are automatically provided the total time they have spent meditating or listening (time from the first meditation or podcast to present), total sessions, and longest streak after each use. To promote competence, the Calm and podcast apps include a reminder that participants can set in the app that will send them a push notification to meditate or listen to a podcast at a set time each day. To promote relatedness, the final feature of the Calm and podcast apps includes *Share Your Stats*, which allows participants to share summaries of their meditation or podcast listening use with others via SMS text messages, emails, or social media (eg, Facebook, Pinterest, or Instagram).

In addition to the above app features, EZTexting (a commercial SMS text messaging–based platform) will be used to send a brief, automated phone call, email, or SMS text message (based on participant preference) at the beginning of the week to encourage continued participation, with the message and conversation grounded in the 3 tenets of SDT (ie, autonomy, competence, and relatedness). Participants who are not meditating or listening to the podcast episodes for at least 10 minutes per day or 70 minutes per week for >3 consecutive days will receive a midweek phone call, email, or text (based on participant preference) through EZTexting. Participants could opt out of these follow-up messages at any time during the intervention. Research staff will track adherence information throughout the study and determine whether the patterns of use are similar across arms. Furthermore, both the Calm intervention and podcast will automatically show the user their time spent in meditation or listening for the day, the total time since the start of the study, and the longest streak as a reinforcing self-management technique. Finally, the Calm intervention and podcast also include reminder tools in which participants can set reminders on their phones to participate [73]. All study participants will receive a monetary incentive for completion of the baseline (US $15), postintervention (US $25), and follow-up (US $35) assessments, including blood draws, for a total incentive opportunity of US $75 paid in one lump sum at the end of the study if all 3 outcome time points are completed.

### Study Outcomes and Measures

#### Overview

Table 3 presents the study outcomes and measures. All study participants will be asked to complete web-based questionnaires via REDCap at 3 time points: baseline (week 0), postintervention (week 8), and follow-up (week 20). In addition, participants will complete a weekly web-based sleep diary via REDCap from weeks 1 to 8 and at the end of week 20. Participants will also complete a weekly log via REDCap regarding their satisfaction with the study and whether there have been any changes in their cancer therapy, sleep medication and supplement use, and disease status. The weekly satisfaction logs will specifically include questions to assess the participants’ overall perceptions of their meditation or podcast sessions. If the participant meditates or listens to a podcast, they will be asked about their likes, dislikes, facilitators, and barriers. If they do not meditate or listen to a podcast, they will be asked what would help them.

**Table 3:** Summary of data collection.

Data, outcomes, and measures				Week 1		Weekly		Week 8		Week 20
**Aim 1**										
	**Sleep disturbance (subjective)**									
		Insomnia Severity Index	✓				✓		✓	
		PROMIS^a^ Sleep Disturbance	✓				✓		✓	
		PROMIS Sleep-Related Impairment	✓				✓		✓	
		Sleep diary			✓				✓	
	**Sleep disturbance (objective)**									
		Actiwatch			✓^b^				✓	
**Aim 2**										
	**Inflammation**									
		Serum cytokines and proteins (TNF-α^c^, interleukin-6, interleukin-8, and CRP^d^)	✓				✓		✓	
	**Fatigue**									
		PROMIS Adult Cancer Fatigue	✓				✓		✓	
	**Depression**									
		PROMIS Depression	✓				✓		✓	
	**Anxiety**									
		PROMIS Anxiety	✓				✓		✓	
**Demographics**										
	**Biological sex**									
		Self-report	✓							
	**Age**									
		Self-report	✓							
	**Race and ethnicity**									
		Self-report	✓							
	**Income**									
		Self-report	✓							
	**Education**									
		Self-report	✓							
	**Marital status**									
		Self-report	✓							
	**Occupation**									
		Self-report	✓							
**Disease and treatment variables**										
	**Comorbidity condition**									
		Charlson Comorbidity Index	✓							
	**Blood counts**									
		Complete blood count	✓				✓		✓	
	**Sleep medications**									
		Self-reported			✓				✓	
	**Cancer therapy changes**									
		Self-reported			✓				✓	
	**BMI**									
		Self-reported height and weight	✓							
**Fidelity and process**										
	**Meditation participation**									
		Self-report log			✓					
		Calm-provided data			✓					
	**Podcast participation**									
		Self-report log			✓					
		App-provided data			✓					
	**Satisfaction**									
		Self-report log			✓					
	**Check-ins and reminders**									
		Email, SMS text message, and phone call			✓					

^a^PROMIS: Patient-Reported Outcomes Measurement Information System.

^b^Assessed daily during the intervention period (weeks 0-8) and for a 7-day period at week 20.

^c^TNF-α: tumor necrosis factor-α.

^d^CRP: C-reactive protein.

#### Demographics

Demographic characteristics will be self-reported at baseline and will include biological sex, age, race, ethnicity, marital status, income, education, and occupation.

#### Clinical Data

Clinical characteristics will include a comprehensive assessment of cancer-specific disease information (eg, comorbidities and complete blood count); other comorbidities (Charlson Comorbidity Index) [74]; self-reported weight and height; and current pharmacological treatment information, including sleep medication.

#### Primary Outcomes (Sleep)

The primary outcome of changes in sleep disturbance will be assessed using the Insomnia Severity Index (ISI). The ISI, a 7-item measure of reported insomnia symptom severity over the past 2 weeks, has been validated among patients with cancer (Cronbach α=.90) [75]. Total scores range from 0 to 28, with scores of 8 to 14 and ≥15 indicating subclinical and moderate to severe insomnia symptom severity, respectively [75]. An ISI change score >7 is considered a clinically meaningful response to treatment [75]. Sleep will secondarily be assessed using the National Institutes of Health (NIH) Patient-Reported Outcomes Measurement Information System (PROMIS) Sleep Disturbance Short Form 8b, PROMIS Sleep Impairment Scale, actigraphy, and sleep diaries. The PROMIS Sleep Disturbance Short Form 8b comprises 8 items assessing various aspects of sleep on a 1 to 5 Likert scale. The measure generates a total summed score, which is then converted to a standardized *t* score for analysis [76]. The NIH PROMIS Sleep Disturbance Short Form 8b is a reliable and valid measure for assessing sleep disturbance in patients with cancer (Cronbach α=.86) [76]. The PROMIS Sleep-Related Impairment Short Form 8a comprises 8 items assessing various aspects of sleep-related impairment (self-reported perceptions of alertness, sleepiness, and tiredness during usual waking hours and perceived functional impairments during wakefulness associated with sleep problems or impaired alertness) on a 1 to 5 Likert scale. The measure generates a total summed score, which is then converted to a standardized *t* score for use in analyses [77,78]. The PROMIS Sleep-Related Impairment Short Form 8a is a reliable and valid measure for assessing impairments associated with sleep across a range of healthy and patient populations reporting sleep disturbance and sleep impairments (Cronbach α=.85-.91) [77,78].

Sleep will also be assessed objectively via wrist-worn actigraph, specifically, via an Actiwatch Spectrum Plus device (Philips Respironics, Inc). Participants will wear the Actiwatch daily throughout the 8-week intervention period, mail it back to research personnel after week 8 (the Actiwatch has a maximum battery life of 68 days), and receive an Actiwatch to wear again for a 7-day period during the follow-up period or week 20. The Actiwatch Spectrum Plus is a valid, reliable, and objective assessment of sleep to be worn on the nondominant wrist 24 hours a day and 7 days a week. This assesses the motion and ambient light data used to analyze the sleep and circadian rhythm parameters. Actigraphy data of ≥5 nocturnal periods recorded per week during the intervention period (ie, week 0-8) and during the 7-day follow-up or week 20 will be considered valid, and the actigraphy data will be binned weekly. The sleep-wake status in each 30-second epoch during the rest intervals will be determined with the Actiware version 6.1.2 (Phillips) scoring algorithm at the medium wake sensitivity threshold. The Actiware software contains automatic off-wrist detection and scoring. All main and minor rest intervals will be verified, with participants entering the rest interval onset and offset times, which are marked at the epoch level when the participant pushes a corresponding button on the device. If these event markers are absent, then the rest intervals will be verified using a daily sleep diary.

Finally, sleep will be further assessed using the Consensus Sleep Diary (CSD). The CSD will be used to assess reported bedtimes and wake times to determine the sleep and wake windows of the main and minor sleep periods [79]. Similar to the actigraphy data, sleep data from the CSD will also be binned weekly. The following night-level and averaged sleep metrics will be derived from both sleep diaries and actigraphy: bedtime, wake time, time in bed, total sleep time, sleep onset latency, wakefulness after sleep onset, number of awakenings, and sleep efficiency ([total sleep time/time in bed]×100%). Sleep efficiency from both sleep diaries and actigraphy will be the main secondary sleep outcome of interest.

#### Secondary Outcomes

Inflammatory biomarkers with serum cytokines and proteins (TNF-α, IL-6, IL-8, and CRP) will be objectively assessed from participant blood samples. The participants’ blood draw instructions will include the address of the nearest Quest Diagnostics Patient Service Center (there are approximately 2200 centers located across the United States). Participants will be instructed to have their blood *drawn in the morning before 10 AM* within 2 weeks of notifying research personnel that they received their package and to repeat their blood draw at postintervention (8 weeks) and follow-up (20 weeks) time points. Participants will not need to fast and follow any other *day-of* procedures. In our prior work, we had high compliance with this method (92.6% at baseline and 70.4% at the postintervention time point) despite offering no incentives for completion of the blood draws [80]. A certified phlebotomist will perform all blood tests, drawing 3 mL of blood. Blood will be transferred into a plastic screw-cap vial and stored on cold packs. The Patient Service Center will mail the drawn blood to the Clinical Laboratory Improvement Amendments–-certified laboratory at the Quest Diagnostics Nichols Institute (San Juan Capistrano, California) for analysis. There will be no cost to the participants. Quest Diagnostics will provide test results to research staff via a secure web-based platform.

Fatigue and emotional distress (ie, anxiety and depression) will be assessed using the NIH PROMIS Adult Cancer Fatigue 7a (7 items), NIH PROMIS Emotional Distress-Anxiety Short Form 7a (7 items), and NIH PROMIS Emotional Distress-Depression Short Form 8a (8 items). Each of these measures asks participants questions on a 1 to 5 Likert scale and generates a total summed score, which is then converted to a standardized *t* score for use in the analyses. These NIH PROMIS instruments are reliable and valid measures of fatigue (Cronbach α>.86), anxiety (Cronbach α=.90), and depression (Cronbach α=.91) in patients with cancer [76].

### Statistical Analysis

#### Sample Size and Power

All estimates presented here are conservatively based on a generalized linear mixed model analytic approach, in which the focal effect is an arm (Calm vs podcast) × time (baseline vs 8 or 20 weeks) interaction. A generalized linear mixed model approach affords lower power than the preferred analysis of covariance (ANCOVA) approach, which will be pursued if the model’s homogeneity of effect assumption is met (see the *Analytic Approach* section). Assuming a sample SD for ISI scores of 6.0 (derived from the literature on sleep disturbance among patients with cancer [81-85]) and Cronbach α=.05, a complete-case sample size of 28 (n=14 pee arm) would afford a power of 0.80 to detect a clinically meaningful between-arm (Calm vs podcast) differences in ISI scores of >7 points (ie, a standardized between-arm difference of Cohen *d*=1.33). However, anticipated effects on other sleep measures and measures of well-being are generally much more modest; accordingly, the study sample size was determined based on anticipated intervention effects on these outcomes rather than those on the ISI. In our previous work, we found moderate effects of meditation on self-reported psychological well-being (aim 2; Cohen *d*_s_=0.31-0.44), self-reported sleep disturbance assessed via the PROMIS Sleep Disturbance questionnaire (Cohen *d*=0.47), and inflammation biomarkers (Cohen *d*_s_=0.41-0.50) [39]. The meta-analysis by Duong et al [86] suggests somewhat stronger effects of mindfulness (standardized mean difference 0.50) and relaxation (standardized mean difference 0.94) interventions on fatigue. Assuming an effect size typical of those observed across these various outcomes (Cohen *d*=0.45), a conservative estimate of the pre-post correlation for outcome scores (*r*=0.30) and Cronbach α=0.05, a complete-case sample (at 8 weeks) of 220 (n=110 per arm) should afford a power of 0.80 to detect significant between-arm differences in secondary outcomes (and power >0.99 to detect a clinically meaningful difference in ISI scores). Anticipating up to 20% attrition at 8 weeks, we will enroll and randomize 276 participants (n=138 per arm). For aim 3, anticipating up to 30% attrition by week 20 (yielding 194 complete cases; n=97 per arm), tests of intervention effects should have power=0.80 to detect the effects of Cohen *d*≥0.48. Multivariable linear regression analyses of potential dose-response effects on outcomes will take two forms: (1) analysis of all available cases with a dose coded as 0 for the podcast group and (2) analysis using data from the Calm group only. These analyses should have a power of 0.80 to detect small (*r^2^*≥0.039) and moderate (*r^2^*≥0.076) linear dose-response effects.

#### Analytic Approach

For preliminary analyses, univariate and bivariate statistics and plots will be used to examine the distributions of and associations between study variables, identify potential multivariate outliers and influential cases, explore patterns of missingness, and check for systematic between-arm differences in baseline participant characteristics. Before addressing the study aims, we will estimate multivariable linear regression models predicting week 8 and week 20 values of each outcome from the main effects of the treatment arm (arm) and the corresponding baseline value of the outcome (baseline) and an arm × baseline interaction term. If the interaction is not significant, an ANCOVA-type approach (described in the following paragraphs) will be used. However, if this interaction is significant, a generalized linear mixed model approach will be used, with repeated measurements of outcomes treated as nested within the participants. Preliminary analyses will inform the choice of link function (eg, natural log) and error distributions (eg, negative binomial) or the transformation of outcome scores used in the primary analyses. R (version 4.1.2) will be used for all analyses and plots.

To assess intervention effects on sleep quality and sleep-related impairment, we will use multivariable linear regression models (following an ANCOVA-type approach), predicting week 8 values on the ISI and secondary sleep outcomes, including the PROMIS Sleep Disturbance questionnaire and Sleep-related Impairment Scale, and sleep efficiency measured via sleep diaries and actigraphy from the study arm (Calm v podcast), adjusting for corresponding baseline values and relevant background covariates (eg, prestudy sleep medication dosage and BMI). These analyses, which will follow a strict intent-to-treat approach, will be supplemented by parallel analyses in which week 8 outcome values will be predicted from dose (minutes of meditation use), with the dose coded as 0 for the podcast arm, while adjusting for baseline outcome measure values and background covariates. These supplemental analyses will address heterogeneity in intervention exposure without including highly collinear arm and dose terms in the same models.

To address the hypotheses regarding the intervention effects on inflammatory markers (TNF-α, IL-6, IL-8, and CRP), fatigue, anxiety, and depressive symptoms, we will estimate models parallel to those estimated for sleep measures, with week 8 outcome values predicted based on the arm, adjusting for corresponding baseline values on outcome measures and relevant background covariates.

To address the hypotheses regarding longer-term intervention effects at week 20, we will use analytic approaches parallel to those to assess differences in change at week 8, with outcomes being predicted from arm or dose (minutes spent meditating), adjusting for corresponding baseline outcome values and background covariates.

To address the hypotheses regarding associations between dose and sustained meditation use (number of weeks with ≥4 days of meditation use) and longer-term change in outcomes within only the Calm intervention arm, we will examine bivariate plots and estimate a variety of multivariable regression models (eg, polynomial linear regression, piecewise linear function, and generalized additive models) to understand the forms of the associations of dose and sustained use and change in outcomes.

If preliminary analyses indicate significant arm × baseline interactions, we will use (instead of an ANCOVA approach) a generalized linear mixed model approach in which a cross-level arm (Calm vs podcast) × time (eg, baseline vs week 8) interaction term is the focal effect for predicting repeated measurements of the outcome measure. This interaction term will capture between-arm differences in changes from baseline to week 8 (or week 20). Differences in change as a function of the intervention dose will be captured using dose × time interaction terms. Although the generalized linear mixed model approach generally does not provide as much statistical power as the ANCOVA approach, generalized linear mixed models (estimated using maximum likelihood) have the advantage of drawing on the strength of all available covariate and outcome data to produce unbiased parameter estimates and SEs in the presence of outcome data that are missing at random.

All preliminary analyses will be supplemented by analyses exploring the interactions between biological sex (male vs female) and the focal effect in each model (eg, sex × arm in ANCOVAs or sex × arm × time in generalized linear mixed models) to determine whether these effects differ by sex. Furthermore, we will conduct sensitivity analyses by examining associations between time of day (eg, proportion of meditation bouts occurring within 2 hours of bedtime) and the proportion of sessions using nonmeditative content and outcomes at 8 and 20 weeks, adjusting for baseline outcome values. As these variables will be highly collinear with the study arm because podcast users will not have any recorded in-app meditation use, sensitivity analyses gauging the potential impact of these variables will parallel the dose-response analyses described previously. Further sensitivity analyses will be conducted to assess the potential impact of (1) changes in the chronic hematological cancer treatment regimen and (2) sleep disorder diagnoses that occur after randomization. For these, analyses of intervention effects will be repeated, excluding data from individuals who experience either of these events, and the results will be compared with those from the full intent-to-treat sample. To maintain the type I error rates at a nominal Cronbach α=.05, we will use *P* values adjusted using the Hommel approach [87].

#### Missing Data

If an ANCOVA-type approach is pursued, we will use multiple imputation to impute missing data by including all the variables from a given analytic model (ie, the variables with missingness will be included in the imputation model) in the imputation model, as well as potentially important auxiliary variables that are predictive of missingness or found to be associated with variables that have missingness [88]. With the *mice* package in R, we will use the fully conditional specification to generate 100 imputed data sets to be analyzed, as described previously [89]. Although generalized linear mixed models are robust to missingness in the response variable, data from participants with incomplete data on ≥1 covariate will be excluded from the analyses. Therefore, where warranted, we will use multiple imputation to generate imputed data sets to be analyzed via linear mixed models.

## Results

This project is funded by the NIH National Cancer Institute (R01CA262041). The projects kicked off in April 2022, and study recruitment is scheduled to begin in October 2022, with the total project duration lasting 5 years. We anticipate that we will be able to achieve our enrollment goal of 276 patients with chronic hematological cancer within the allotted project time frame.

## Discussion

### Principal Findings

The purpose of this study is to leverage an academic-industry partnership with the consumer-based app Calm to rigorously evaluate Calm for the effective delivery of a meditation intervention to patients with chronic hematological cancer via a 20-week RCT. Specifically, in this study, we aimed to (1) test the efficacy of daily meditation (≥10 minutes per day) delivered via Calm compared with a health education podcast control group on self-reported sleep disturbance, sleep impairment, and sleep efficiency; (2) test the efficacy of daily meditation (≥10 minutes per day) delivered via Calm compared with a health education podcast control group on inflammatory markers, fatigue, and emotional distress; and (3) explore the sustained effects at 20 weeks from the baseline of Calm in patients with chronic hematological cancer. This study will be the first RCT to determine the effects of a consumer-based mobile meditation app on sleep disturbances in patients with chronic hematological cancer. If our findings demonstrate a significant clinical impact on sleep disturbance in patients with chronic hematological cancer, we will have an inexpensive, easily accessible, nonpharmacological intervention that can readily be prescribed by cancer care providers for sleep disturbance in chronic hematological cancer. Our rigorous RCT will fill a knowledge and rigor gap regarding the delivery of smartphone-based meditation as an intervention for sleep and provide new data on sustained effects.

### Comparison With Prior Work

There is limited understanding of the effects of meditation on sleep-related outcomes in patients with cancer, with no research to date examining these outcomes in hematological cancers (1.9 million in the United States) [28,57]. Existing research using apps has focused on emotional measures or cancer-specific biological changes as outcomes but not sleep disturbance [90-94] in survivors of solid tumors with minimal residual disease or those with short survival times (eg, patients with metastatic solid tumors). The different and distinct long-term disease courses of patients with chronic hematological cancer and the associated maintenance therapy toxicities and symptoms warrant innovative interventions that support the self-management of symptoms [3]. Patients with chronic hematological cancer are often geographically dispersed, with substantial variability in their proximity to cancer centers because of the rarity of certain subtypes (eg, MPNs) [95], limiting opportunities for community-based local programs to help manage symptoms. Remote delivery of meditation via a mobile app will improve reach and scalability in this understudied population.

In addition, previous studies on meditation in cancer have focused primarily on White women with breast cancer [57]. This study presents an opportunity to study meditation in older adults, men, and minorities who present with chronic hematological cancer. Our prior successful recruitment strategies for patients with cancer will specifically recruit representative samples from these populations. Furthermore, we previously established that Calm is acceptable and appealing to a wide population group, which can expand the reach of traditional research interventions. For example, in a cross-sectional survey of Calm subscribers (N=12,151), 20% were male, 19% were people of color, and 15% were older adults (aged >65 years).

Prior studies using meditation to reduce sleep disturbance in patients with cancer did not have follow-up periods beyond 12 weeks [53]. In the 2 meditation-based interventions conducted in patients with chronic hematological cancer, both were delivered for a very short period (ie, 5 sessions of 1 hour per week). No product could be used continually and accessed beyond study completion [58,59]. As most evidence-based apps are developed within the context of research studies and are not sustained at the end of the study [96], the scalability and viability of the widespread distribution of mobile app–based interventions in these studies are not possible. As Calm is consumer based, the intervention never truly *ends*. Calm is available and can be used whenever a patient desires or feels it is needed (eg, when they feel overwhelmed, fatigued, anxious, or lying down to go to sleep). The patient can choose when and how frequently Calm is used, increasing the likelihood that patients will use Calm to alleviate sleep disturbance in the long term. This study can uniquely measure and explore time and dose effects and will advance the evidence for a consumer-based meditation app that patients with chronic hematological cancer can use for symptom self-management in the long term. This research will contribute to broader public health efforts by providing researchers and clinicians with an evidence-based commercial product to improve sleep in the long term in an underserved and understudied population with cancer and a high incidence of sleep disturbance.

### Future Directions

If it is demonstrated that Calm is an efficacious intervention for patients with chronic hematological cancer, future work will aim to disseminate Calm as an evidence-based option for health care providers to recommend or prescribe to patients with chronic hematological cancer. Consideration will be made to apply for a NIH Small Business Innovation Research grant to help with the dissemination of Calm or to receive Food and Drug Administration approval for Calm as a nonpharmacological intervention for the treatment of sleep in patients with chronic hematological cancer.

### Conclusions

In summary, this study will contribute to the mindfulness and cancer literature at large, with a unique focus on filling the much-needed gap by examining remote mindfulness-based interventions in patients with chronic hematological cancer.

## Abbreviations

ANCOVA: analysis of covariance
CBT-I: cognitive behavioral therapy for insomnia
CRP: C-reactive protein
CSD: Consensus Sleep Diary
IL: interleukin
ISI: Insomnia Severity Index
MBSR: mindfulness-based stress reduction
MPN: myeloproliferative neoplasm
NIH: National Institutes of Health
PROMIS: Patient-Reported Outcomes Measurement Information System
RCT: randomized controlled trial
REDCap: Research Electronic Data Capture
SDT: self-determination theory
TNF-α: tumor necrosis factor-α

## Data Availability

All data gathered in this study will be kept private. A reasonable request can be made by researchers and physicians to have access to deidentified data when data collection is complete.
